# Supplementary material for: Thermo-responsive gels that absorb moisture and ooze water
Source: Nat Commun. 2018 Jun 13;9:2315. doi: 10.1038/s41467-018-04810-8 (PMC5998054; doi:10.1038/s41467-018-04810-8)
Supplement: Supplementary file 1 — Supplementary Information [file 41467_2018_4810_MOESM1_ESM.pdf]

Thermo-Responsive Gels That Absorb Moisture and Ooze Water

Matsumoto et. al.

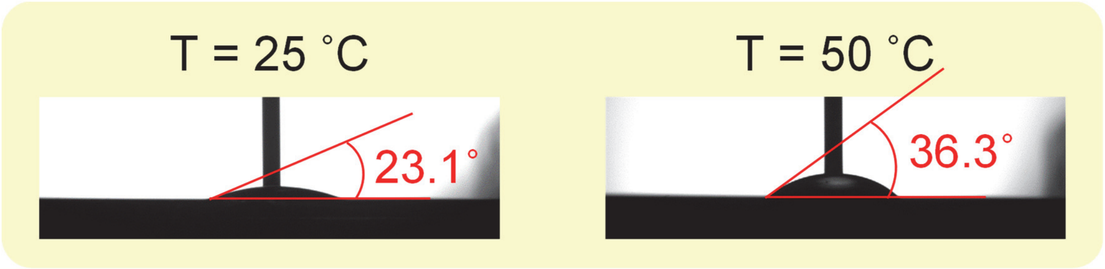

Supplementary Figure 1. Water contact angle on IPN gel below and above its LCST. The contact angles of water on the PNIPAAm/Alg IPN gel were measured at 25 °C (below LCST) and 50 °C (above LCST).
