# Supplementary material for: Thermo-responsive gels that absorb moisture and ooze water
Source: Nat Commun. 2018 Jun 13;9:2315. doi: 10.1038/s41467-018-04810-8 (PMC5998054; doi:10.1038/s41467-018-04810-8)
Supplement: Supplementary file 3 — Description of Additional Supplementary Files [file 41467_2018_4810_MOESM3_ESM.pdf]

Description of Additional Supplementary Files

File Name: Supplementary Movie 1
Description: Water oozing behaviour of the PNIPAAm/Alg IPN gel during heating at 50 °C after moisture absorption.
